# Changing the tide in vitamin D testing: An 8-year review of a demand management approach

**DOI:** 10.11613/BM.2024.010401

**Published:** 2024-02-15

**Authors:** Janne Cadamuro, Ursula Huber-Schönauer, Cornelia Mrazek, Lukas Hehenwarter, Ulrike Kipman, Thomas K. Felder, Christian Pirich

**Affiliations:** 1 Department of Laboratory Medicine, Paracelsus Medical University Salzburg, Salzburg, Austria; 2 Department of Nuclear Medicine and Endocrinology, Paracelsus Medical University Salzburg, Salzburg, Austria; 3 High End Statistics, College of Education, Salzburg, Austria

**Keywords:** demand management, preanalytical phase, laboratory order, vitamin D

## Abstract

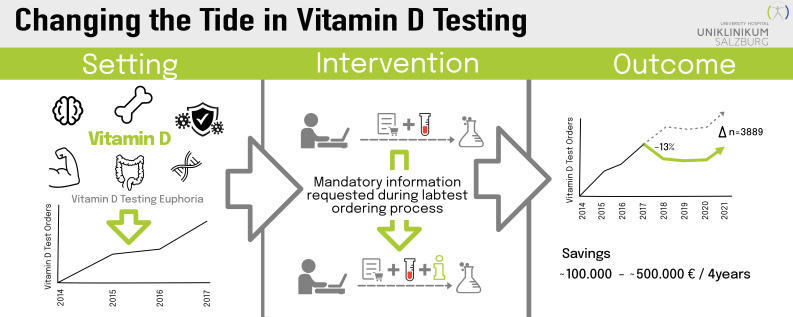

## Highlights

• Adding a mandatory accompanying information in the Vitamin D ordering process led to a significant reduction overall orders

• Given that about one third of mandatory information was incorrect, we assume that the sheer obstacle is sufficient to reduce test numbers, regardless of its type or content

• We estimated potential cost savings ranging from € 101,292 to € 516,704 for a four-year period

## To the editor,

in the past decade(s) the role of vitamin D (VitD) has been claimed to play key roles in processes and metabolisms other than the long since known calcium phosphate regulation, like blood pressure regulation, blood sugar control, parathyroid hormone regulation, immunomodulation, promotion of innate immunity and many others (*1*). It is estimated that 1 billion people worldwide have VitD deficiency or insufficiency, prevalence varying by country and age. Consequently, this has led to a substantial increase in VitD testing.

**Figure 1:**
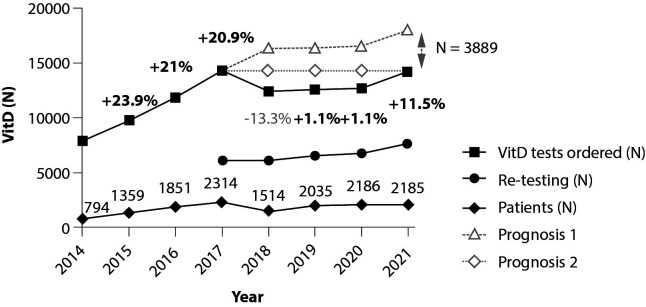
Actual and estimated development of vitamin D (VitD) orders over time. Black squares - overall VitD orders from the entire hospital (in- and outpatients). Black circles - number of re-tests among the total VitD tests (patients with preceding Vit-D results within the year prior to the evaluated test result). Black rhombus - number of patients the VitD results originated from. Trajectory 1 (grey triangles) is estimating a similar annual increase as the actual numbers, while trajectory 2 (grey rhombus) assumes a saturation effect of VitD orders. Percentages represent the relative difference of orders as compared to the preceding year. Numbers above the patient-line represents the according number of patients in that year.

Apart from medical consequences of VitD testing in deficient patients, a number of unsolved issues remain. On one hand, the analytical methods are not yet standardized, leading to differing results and reference ranges among laboratories (*2*). On the other hand, VitD test ordering behaviors are frequently disrespecting existing guidelines, resulting in a considerable overuse of laboratory resources as well as potential unnecessary follow-up diagnostics or treatments. Reported numbers of inadequate VitD testing range as high as 57% of all VitD tests performed, varying throughout healthcare settings (*3*-*5*). Similarly, VitD orders increased also in our hospital annually by about 20% (Figure 1).

In an effort to streamline test requests in concordance to current recommendations and after consensus with clinical specialists, a mandatory additional information regarding the type of order was queried upon VitD test ordering, starting on 31st of December 2017. In the local computerized physician Order Entry (CPOE) system (ORBIS, Agfa HealthCare, Bonn, Germany) a selection between „ initial measurement” and “follow-up measurement” had to be made during ordering. This demand management strategy was implemented only after consensus with clinical specialists. The implementation itself was not communicated *via* official channels such as the intranet. Users were confronted with the new mandatory step when ordering vitamin D testing after 2017. Educational strategies in other types of diagnostic testing was done many times prior to this implementation and have been proven insufficient, probably due to the hospital size and personnel turnaround.

In order to quantify the impact of this intervention on overall VitD requests as well as on financial savings, anonymized retrospective VitD measurements (Architect i 2000SR 25-OH Vitamin D, Abbott Diagnostics, Wiesbaden, Germany) from in- and outpatients four years before (N = 43,978) (64% female, age 58 years (0-102)) and after (N=51,935) (66% female, age 54 years (0-105)) this intervention (2014 to 2021) were retrieved from the local laboratory information system (LIS, GLIMS, Clinisys Deutschland GmbH, Walluf, Germany) at the University Hospital of Salzburg, a 1700-bed tertiary care center serving 650,000 inhabitants. We then calculated the number of total VitD tests performed annually for the entire hospital and found a 13% decrease in 2018, quite stable numbers in 2019/2020 and an 11% increase in 2021. Although we cannot exclude other influencing variables, we assume that these changes are directly associated with the aforementioned intervention.

To estimate the overall number of tests saved by the intervention, we then calculated two different prognostic developments of VitD orders, assuming that no intervention had taken place. For the first calculation we assumed an indefinite incline in orders, by applying a best fit regression to the numbers from the years 2014 to 2017 (linear regression; R2 = 0.996; N = 2137.2 x Year – 4,296,532.10). For the second estimation, we assumed a saturation effect of VitD test, based on the number of tests in the year 2017 (Figure 1). Subsequently applying two different reimbursement systems used in Germany and Austria (Gebührenordnung für Ärzte - https://abrechnungsstelle.com/goae/goae-abschnitt/m/; Einheitlicher Bewertungsmaßstab der kassenärztlichen Bundesvereinigung https://www.kbv.de/html/online-ebm.php), we found overall savings ranging from € 286,194 to € 516,704 for prognosis 1 and from € 101,292 to € 182,876 for prognosis 2.

To evaluate whether the primary/initial testing (*i.e.* VitD testing in cases *without* preceding results within the year prior to the evaluated test result) or of the retesting (*i.e.* VitD testing in cases *with* preceding results within the year prior to the evaluated test result) habits were impacted by our intervention, we calculated the number of re-tests. We found that especially initial testing was impacted by our intervention in comparison to re-testing, indicating that inappropriate testing in patients without the risk of VitD deficiency could potentially be averted (Figure 1). However, as we have access only to our own Electronic Health Record (EHR) system, we cannot account for eventual VitD controls performed outside of our hospital (*e.g.*, by the general practitioner), potentially altering initial and re-testing numbers. Unfortunately, since we were unable to retrieve reliable information on the clinical signs and symptoms or the supplementation state of the patients in which VitD testing was requested, we could not perform further in-depth evaluation on medical inappropriateness.

In addition, we evaluated the compliance of ordering personnel to the mandatory input, by calculating the correctness of entries such as „Follow-up measurement“ with no previous results or „Initial measurement“ with existing previous results. In the former case, the result could be biased by the non-inclusion of external VitD results. We found that about one third of this compulsory information was provided incorrectly, a circumstance many laboratory specialists, especially from large hospitals, know too well, namely that mandatory information in great parts is entered blindly to get to the ordering sheet, irrespective of the fact if this information is true or not. In this line of thought, one could hypothesize that the “correct” statements were in part only correct by chance. Either way, this finding indicates that the implementation of an additional mandatory step during the ordering process seems sufficient to decrease overall VitD ordering numbers, regardless of the type or content of such a step.

We conclude that applying a simple mandatory obstacle in the CPOE system may be sufficient not only to prohibit the further incline, but to reduce overall VitD testing frequency, subsequently leading to a considerable economic savings.

We want to emphasize that a close collaboration with all stakeholders during such a process is mandatory in order to increase the probability of a successful intervention and to maintain or improve a good relationship to clinicians.

## Data Availability

The data generated and analyzed in the presented study are available from the corresponding author on reasonable request.
